# Undiagnosed Depression among Hypertensive Individuals in Gaza: A Cross-sectional Survey from Palestine

**DOI:** 10.4314/ejhs.v31i2.17

**Published:** 2021-03

**Authors:** Khalid Jamal Khadoura, Elham Shakibazadeh, Mohammad Ali Mansournia, Yousef Aljeesh, Akbar Fotouhi

**Affiliations:** 1 Department of Epidemiology and Biostatistics, School of Public Health, Tehran University of Medical Sciences-International Campus, Tehran, Iran; 2 Department of Nursing, Faculty of Medical Sciences, Israa University-Gaza, Palestine; 3 Department of Health Education and Promotion, School of Public Health, Tehran University of Medical Sciences, Tehran, Iran; 4 Department of Epidemiology and Biostatistics, School of Public Health, Tehran University of Medical Sciences, Tehran, Iran; 5 Faculty of Nursing, Islamic University of Gaza, Gaza, Palestine

**Keywords:** Hypertension, Depression, Prevalence, Medication adherence

## Abstract

**Background:**

The aim of this study was to estimate the prevalence and to determine the associated factors of undiagnosed depression amongst hypertensive patients (HTNP) at primary health care centers (PHCC) in Gaza.

**Methods:**

A cross-sectional survey was conducted including 538 HTNP as a recruitment phase of a clustered randomized controlled trial. Data were collected through face-to-face structured interview, and depression status was assessed by Beck's Depression Inventory (BDI-II). Data were analyzed by STATA version 14 using standard complex survey analyses, accounted for unresponsiveness and clustering approach. Generalized linear regression analysis was performed to assess associations.

**Results:**

The prevalence of undiagnosed clinical depression was 11.6% (95% confidence interval [CI]: 8.1, 16.3). Moreover, prevalence of 15.4% (95% CI: 10.8, 21.6) was found for mild depression symptoms. We found that non-adherence to antihypertensive medications (AHTNM) (β = 0.9, 95% CI: 0.17, 1.7), having more health-care system support (β = 2.8, 95% CI: 1.6, 3.9) and number of AHTNM (β = 1.5, 95% CI: 0.6, 2.5) remain significantly positively associated with BDI-II score. On the other hand, older age (β = -0.1, 95% CI: -0.2, -0.02), having better social support (β = -6.8, 95% CI: -8.9, -4.7) and having stronger patient-doctor relationship (β = -4.1, 95% CI: -6.9, -1.2) kept significantly negative association.

**Conclusion:**

The prevalence of undiagnosed depression was about one-quarter of all cases; half of them were moderate to severe. Routine screening of depression status should be a part of the care of HTNP in PHCC.

## Introduction

Globally, depression affects 350 million people around the world; it is strongly contributes to the burden of disease, and is expected to increase by 5.7% of global burden of disease and become the second after ischemic heart disease by 2020 (1). Notably, episodes of depression are accompanied with other chronic diseases especially hypertension (2,3). Hypertension affects globally one fourth of adults, and is likely to increase to one third by 2025 (4). People with hypertension and subclinical depression are at extra risk of complications such as cerebrovascular stroke, cardiovascular and kidney diseases (3, 5–7). Moreover, depression represents an important predictor among hypertensive individual whom are non-adherence to their treatment (8).

Many studies demonstrated an increased co-occurrence of depression with hypertension in different countries (9); however, little is known about depression prevalence among hypertensive patients in Gaza Strip (GS). This study seems to be the first study aimed to estimate the prevalence and to determine the associated factors of undiagnosed depression among hypertensive patients attending primary healthcare clinics in GS.

## Methods and Participants

A cross-sectional survey as the recruitment phase of a clustered randomized controlled trial was conducted between 1st August and 30th December, 2018. We recruited 538 hypertensive persons seeking primary health care by two stages cluster random sampling from ten primary health centers. Initially, centers were randomly selected by stratified simple random sampling approach to get two centers from each governorate. Then, participants from each center were proportionally selected through a systematic random sampling method based on eligibility criteria and agreement to take part in the study.

This cross-sectional survey was conducted as the recruitment phase of a clustered randomized controlled trial. All eligible participants who agreed to participate in the study were enrolled regardless of their antihypertensive medication adherence status. Psychological status was initially classified according to the BDI-II score. Later, adherent status to antihypertensive medication was determined. Subsequently, the intervention of the clustered randomized controlled trial was implemented based on the adherent status results.

**Measures and data collection**: Face-to-face interview was used to collect data from participants using a structured questionnaire. The interviews lasted fifteen minutes during the clinic hours (8 am to 2 pm, five days a week). The exposure variables of the study included participants' characteristics (age, gender, marital status, employment, education level), as well as participants' health status variables including smoking status, comorbidities, body mass index (BMI), blood pressure (BP) measurement, and medication adherence status. Other predictors of interest such as social support, relationships between patients and physician and health system support were also assessed. Moreover, depressive symptoms as the outcome variable were assessed using the validated Arabic language Beck's Depression Inventory (BDI-II) (10).

Blood pressure was measured on the right arm using mercury sphygmomanometer after completing the interview; the value was recorded in terms of mm of mercury. BMI was calculated by using the WHO chart based on weight and height. Weight and height were measured by mechanical weighing machine with height rod (Health o meter 402LB Physician Beam Scale, Height Rod).

**Instrument**: The questionnaire consisted of items about the baseline characteristics of participants, clinical health history, adherence status, patient-doctor relationship, healthcare system support, perceived social support and psychological status of depressive symptoms.

Depressive symptoms were assessed by the aid of BDI-II. A 21-item self-report inventory was designed to assess the presence and severity of depressive symptoms. Each item is rated on a four-point Likert-type scale ranging from 0 to 3, based on the severity in the last two weeks. The total score ranges from 0 to 63, with higher scores indicating more severe depressive symptoms. The results were stratified into four groups; BDI-II: 1–13 these ups and downs are considered normal, BDI-II: 14–19 are mood disturbance or mild depression, BDI-II: 20–28 are moderate depression, and BDI-II: more than 29 are severe depression (11). We provided psychological counseling by psychotherapist for those who scored from 11 to 19. Special precautions were taken for those individuals who endorsed having suicidal ideation. Mainly, we involved the family and coordinated for urgent counseling in the related psychological health center. Moreover, all cases scored 20 and above were referred to psychology health centers. Consequently, stratification into two groups was done; considering BDI-II ≥20 who have clinical depression and BDI-II <20 who do not have clinical depression (12,13).

Adherence status was assessed by Morisky, Green, and Levine Adherence Scale (MGL), a known validated and reliable self-report medication adherence score (14). Similarly, medical comorbidities were assessed using Charlson comorbidity index, a validated and widely used weighted-index designed scoring as low and high comorbidities (15). Likewise, social support was assessed by multidimensional Scale of Perceived Social Support (MSPSS); a known valid and reliable questionnaire which measures perceptions of support from three sources: family, friends, and a significant other (16). Arabic translation was performed based on WHO five steps for process of translation and adaptation of instrument (17).

An Arabic validated and reliable version of patient-doctor relationship questionnaire-9 (PDRQ-9) was used to assess the relationship between patients and physicians (6,18). Likewise, healthcare system support questionnaire was used with little modifications (18, 19). The whole Arabic questionnaire content and face validity were reviewed by panel of experts. Required changes were made to clarify any ambiguity and to ensure comprehension of Palestinian participants after pilot study, detailed information about the questionnaire are available in our published article elsewhere (20).

**Eligibility criteria**: Palestinian citizens attending Gaza governmental primary health centers, aged above 18 years, registered as hypertension patient since at least one year and taking at least one antihypertensive medication were eligible to participate in the study. Patients with a diagnosis of cognitive impairment, history of depression or being currently on antidepressants as reported by their primary care physician were excluded from the study.

**Data analysis**: Standard complex survey data analysis method was performed by STATA version 14. We accounted for clustering and unresponsiveness using STATA PSU option and unequal probability of selection using sample weight variable analysis and post stratification weight for each age and sex group strata. Furthermore, since the BDI-II score did not follow normality assumption, Generalized Linear Model (GLM) with Gaussian family and log link was run. Moreover, linearized standard error which is quite robust to non-uniformity of variance was used.

Data were described using descriptive statistics; categorical variables were compared using the Chi-squared test. After checking assumption of linear regression, univariable analysis followed by multiple linear regression were performed to assess the association between depression status and all other independent variables including participants' characteristic. Statistically significant variables were included in multiple regression analysis model based on 0.1 level. However, variables had been excluded by backward stepwise elimination method.

**Ethics approval and consent to participate**: Prior to conducting this research study, ethical approval from Tehran University of Medical Sciences Ethical Committee (code number: IR.TUMS.SPH.REC.1396.4828) was obtained. Approvals from the Palestinian Health Research Council(PHRC/HC/322/18) and Research Committee at the Palestinian Ministry of Health were also obtained. Purposes of the study were explained to participants, and they were reassured about confidentiality of data. Each participant was asked to sign a consent paper prior to participation.

## Results

**Participants' characteristics**: Five hundred and thirty-eight participants were included in the survey from the five governorates of Gaza Strip by response rate of 96%. The overall mean age was 57.1 years (95% confidence interval [CI]: 55.9, 58.2) and more than half of them (60.9%) were females. In addition, the majority of them were literate (90.2%), married (90.4%), unemployed (86.5%), and not smokers (81.4%). Since obesity is known to have BMI more than 30 kg/m^2^, it was most common among study population as BMI mean reported 32.3 (95% CI: 31.9, 32.6). Moreover, more than half of the participants (57.2%) had been diagnosed with hypertension for more than five years with a mean of 8.44 years (95% CI: 7.3, 9.4). Almost, two third of them (64.4%) were treated with only one antihypertensive medication once a day (64.8%); however, 35.6% were treated with two or more medications with a frequency of twice or more (35.2%). Only 14.43% of them were classified with two or more comorbidities. In addition, the participants reported a mean of depression status based on BDI-II score of 10 (95%CI: 8.4, 11.6%) (Table 1).

**Table 1:** Participants characteristics (n= 538)

Categorical Variable	Percentage	95% confidence interval	
Age groups (years)			
28–39	06.98	02.9	15.4
40–59	49.71	40.5	58.9
≥60	43.31	30.7	56.7
Gender			
Male	39.05	30.9	47.8
Female	60.95	52.1	69.08
Marital status			
Married	90.43	83.0	94.8
Single	01.87	0.85	4.1
Divorced	01.21	0.50	2.8
Widowed	06.49	2.8	14.1
Employment			
Employed	13.53	10.7	16.9
Jobless	27.93	23.2	33.1
Retired	13.18	07.2	22.9
House wife	45.35	36.5	54.0
Level of education			
Illiterate	09.78	05.5	16.7
Elementary school	43.10	38.4	47.9
Secondary school	27.02	24.4	29.7
University education	20.09	19.1	21.1
BMI (kg/m^2^)			
Normal (18.5 to 24.9)	12.18	08.0	18.1
Overweight (25–29.9)	31.30	27.7	35.1
Obese 30	56.51	54.7	58.3
Duration of hypertension diagnoses (years)			
1–5	42.24	31.2	54.1
6–20	52.57	43.1	61.9
>20	05.19	3.3	7.8
Number of antihypertensive medications			
One medication	64.42	59.6	68.9
Two medication	27.50	23.5	31.8
Three and more med.	08.08	5.46	11.7
Frequency of antihypertensive medications taken per day			
Once	64.84	55.7	72.9
Twice and more	35.16	27.0	44.2
Smoking status			
Never	81.42	76.5	85.4
Former	8.78	5.3	14.2
Current	9.79	7.9	12.1
Other comorbidity			
Low	85.57	71.8	93.2
High (≥2 comorbid disease )	14.43	6.7	28.1
**Continuous variable**	**Mean**	**95% confidence interval**	
Age (year)	57.07	55.93	58.21
BMI (kg/m^2^)	32.26	31.90	32.61
Systolic blood pressure (mmHg)	131.64	127.5	135.77
Diastolic blood pressure (mmHg)	83.28	81.42	85.14
Duration of hypertension (year)	8.44	7.35	9.54
Beck's Depression score (BDI-II)	10.04	8.45	11.63

**Depression status**: The BDI-II mean score for all participants was 10 (95% CI: 8.4, 11.6); and they were categorized into three groups: 1) normal (BDI-II 0–13) which had a prevalence proportion of 73% (95% CI: 65.6, 79.3), 2), mild clinical depression (BDI-II: 14–19) with a prevalence proportion of 15.4% (95% CI: 10.8, 21.6), in which they received psychological counseling in the same clinic by psychotherapist; and 3) clinical depression BDI-II ≥20 with a prevalence of 11.6% (95% CI: 8.1, 16.3) of undiagnosed depression cases which all were referred to psychology health centers for further evaluation and treatment (Table 2).

**Table 2:** Depression status prevalence proportion (n= 538)

Variable	Percentage	95% confidence interval
**Normal (0–13)**	72.99	65.64, 79.27
**Mild clinical depression (14–19)**	15.43	10.79, 21.58
**Clinical depression ≥20**	11.57	8.10, 16.29

**Relationship of depression status and predictors**

Under standard complex survey data setting, the Chi-squared test was used to compare categorical variables of depression status and other explanatory variables. Table 3 shows that depression status had relations with the categorical variables: level of education and BMI categories.

**Table 3:** Relation between depression status and participants characteristics

Variable	No Depression % (BDI-II<20)	Depression % (BDI-II>20)	P value (2 sided) Design-based (F)
Age (years)			
28–35	2.21	1.07	0.299
36–50	21.64	3.36	
51–65	44.72	4.09	
> 66	19.86	3.04	
Gender			
Female	35.25	3.78	0.129
Male	53.18	7.79	
Marital status			
Married	8.33	1.05	0.919
Not married	80.1	10.52	
Employment			
Employed	76.83	9.97	0.932
Unemployed	11.58	1.61	
Level of education			
Illiterate	7.82	1.69	0.011
Literate	80.81	9.67	
BMI kg/m^2^			
Normal (18.5 - 24.9)	7.40	0	
Overweight (25–30)	24.94	2.57	0.029
Obese >30	56.08	8.99	
Number of antihypertensive medications			
One medication	57.15	6.40	0.094
Two and more med.	31.28	5.16	
Frequency of antihypertensive medications taken per day			
Once	58.03	6.34	0.196
Twice and more	30.4	5.23	
Smoking status			
Nonsmoker	78.92	11.06	0.298
Smoker	9.51	0.51	
Other comorbidity			
Low	76.2	9.71	0.592
High (≥2 comorbid disease)	12.23	1.85	

Univariable linear regression analysis presented in Table 4 shows that increased BMI (β = 0.11, 95% CI: 0.01, 0.21), being non-adherent to antihypertensive medications (β = 1.3, 95% CI: 0.5, 2.1) and having better healthcare system support (β = 2.8, 95% CI: 1.2, 4.4) were significantly associated with increased BDI-II score. In contrast, older age (β = -1.5, 95% CI: - 0.29, -0.01), longer duration of hypertension (β = -0.12, 95% CI: -0.19, -0.04), stronger patient-doctor relationship (β = -3.7, 95% CI: -6.4, -1.1), and having superior social support (β = -7.1, 95% CI: -9.3, -4.9) were significantly associated with reduced BDI-II score.

**Table 4:** Univariate linear regression analysis of depression status and predictors

Categorical Variable	Coef.	P value (2 sided)	95% conf. interval	
Gender (female)	1.47	0.114	-0.51	3.46
Marital status (married)	-0.12	0.913	-2.83	2.58
Employment (employed)	0.57	0.822	-5.63	6.77
Level of educational (literate)	0.40	0.605	-1.47	2.28
Smoking status (smoker)	-0.03	0.969	-1.86	1.81
Other comorbidity (high)	0.19	0.747	-1.23	1.60
Frequency of antihypertensive medications taken per day (once)	-1.36	0.376	-4.97	2.24
Number of anti-HTN medications (one)	1.22	0.037*	0.11	2.32
Patient-doctor relationship (good)	-3.75	0.015*	-6.39	-1.09
Health-care system support (good)	2.81	0.006*	1.23	4.39
Social support (superior)	-7.07	<0.001*	-9.26	-4.89
Continuous variable				
Age in years	-0.15	0.039*	-0.29	-0.01
BMI kg/m^2^	0.11	0.038*	0.01	0.21
Duration of hypertension	-0.12	0.011*	-0.19	-0.04
Non-adherence status	1.33	0.009*	0.50	2.14
Systolic BP mmHg	-0.03	0.282	-0.11	0.04
Diastolic BP mmHg	0.09	0.154	-.049	0.23

* Statistically significant variables for multivariable analysis

Multivariable linear regression analysis in Table 5 revealed similar results except for increased BMI and duration of hypertension. Thus, non-adherence to antihypertensive medications (β = 0.9, 95% CI: 0.2, 1.7), having more healthcare system support (β = 2.8, 95% CI: 1.6, 3.9) and number of antihypertensive medications (β = 1.5, 95% CI: 0.6, 2.5) remain significantly positively associated with BDI-II score. On the other hand, older age (β = -0.11, 95% CI: -0.20, -0.02), having better social support (β = -6.8, 95% CI: -8.9, -4.7) and stronger relation with physician (β = -4.1, 95% CI: -6.9, -1.2) remain significantly negatively associated with BDI-II score.

**Table 5:** Multivariable linear regression analysis of depression status and predictors

Variable	Coef.	P value (2 sided)	95% conf. interval	
Age in years	-0.11	0.028	-0.20	-0.02
Number of anti-HTN medications	1.54	0.008	0.60	2.48
Non-adherence status	0.91	0.026	0.17	1.66
Patient-doctor relationship (strong)	-4.06	0.015	-6.93	-1.19
Health-care system support (good)	2.76	0.001	1.65	3.86
Social support (superior)	-6.79	0.000	-8.92	-4.66

## Discussion

To best of our knowledge, this is the first cross-sectional survey from Gaza to document the prevalence of undiagnosed depression among hypertensive patients. We found that the prevalence of undiagnosed clinical depression in our hypertensive population is almost higher than what is observed in Norway (6.2%) and South Africa (6%), and lower than in the US(44.9%), China (44.2%), Mexico (57.5%), Pakistan (40.1%), Croatia (29.6%) and Nigeria (26.7%). However, it was similar to that observed in Brazil (12.1%), Ghana (10.5%) and Netherlands (11.4%) (9, 21). In addition, this prevalence was lower than the rate reported in a systematic review and meta-analysis (26.8%) which summarized the prevalence of depression among hypertensive individuals in 41 studies (9).

Unfortunately, very limited data is available from Arabic regional countries. One study from Saudi Arabia found a prevalence of 20.7% (22). Still, no local prevalence was found from other parts of Palestine. Our study showed that the prevalence of depression among hypertensive patients was lower than the prevalence of patients with type two diabetic in the West Bank of Palestine (40%) (23).

The associated factors found by multiple linear regression in this study were: age, number of antihypertensive medication, adherent status, healthcare system support, patient-doctor relationship and perceived social support. Increasing age was found to be an associated factor with increased depressive symptom in other studies (21, 22, 24, 25), although, our results predicted a negative relationship between age and depressive status. An explanation for this could be related to the nature of aged people in Gaza, since they have more acceptance and are adapted to their disease more than younger adults.

However, gender and smoking (21, 22, 24, 26) found to be associated factors in previous studies, did not reach statistical significant level in our study. Longer hypertension duration has been previously found to be a significantly associated factor in an Indian study (26); and was associated factor in our univariable analysis, although it could not reach significant level in multivariable analysis. In addition, in line with what has been found on a systematic review (8), we found a negative relationship between depression and adherence to antihypertensive medications. Furthermore, statistically significant association with number of antihypertensive medication and depression was observed in our study, which was supported by previous findings (24, 27).

In this study, we have investigated the relation of depression status with healthcare system support, patient-doctor relationship and perceived social support. Interestingly, a positive relationship between depression and healthcare system support was found. This could be explained by the frequent visit of the patient to the primary healthcare clinic, and his increased need to its supportive aids. On the other hand, it was a negative relationship with patient-doctor relationship and social support. It is highly believed to be negative relationship between depression and perceived social support since social support is a significant predictor of depression and hypertension treatment programs (28, 29).

We shed the light on the undiagnosed depression cases among hypertensive persons receiving their usual primary health care in primary healthcare clinics. The estimated prevalence proportion of undiagnosed subclinical depression cases was about one-quarter of all cases, in which half of them had moderate to severe depression status. Furthermore, age, number of antihypertensive medication, adherence status, patient-physician relationship, health-care system support and social support were associated with depression status. We suggest periodically screening of depression and adherence status as a part of routine care for hypertensive primary healthcare seekers, particularly younger hypertensive patients. We also call for exploring the ways to promote social support among them.

However, since this is the first study in Gaza Strip which assessed the psychological status among hypertensive patients, several limitations were observed. First, alcohol consumption is an important factor related to depression and adherence and may confound the reported relationships, although it has not been measured in this study. Our justification is that alcohol consumption is illegal and prohibited to be used in Gaza as Muslim citizens, and so it was difficult to get trustful answers from the participants. Thus, more than 98% of them answered no. Indeed, even if he/she was a user he/she would not tell the truth because of fear of legal prosecutions in spite of assurance about data confidentiality. As a result, we decided to omit this variable as a predictor from this study.

The second limitation is that some antihypertensive medications may be related with depression. However, we were concerned only about the number of antihypertensive medications and the frequency and did not discuss the type and classes of the medications.

Another limitation of this study is that the prevalence of depression would be underestimated because we excluded the already known patients with a history of depression or currently on antidepressant medications. In addition, BDI-II is a depression screening tool only and not a diagnostic tool. This will surely affect the true prevalence of depression and therefore this could be another limitation regarding the estimation of the true prevalence. However, BDI-II as a screening tool gave a good impression of the depression prevalence among hypertensive patients and was able to discover many unknown cases that were referred for further diagnostic procedures. Despite the limitations, the findings support the routine screening of depression and medication adherence status as a part of care for hypertensive patients in PHCCs.
